# SpO_2_ and Heart Rate During a Real Hike at Altitude Are Significantly Different than at Its Simulation in Normobaric Hypoxia

**DOI:** 10.3389/fphys.2017.00081

**Published:** 2017-02-13

**Authors:** Nikolaus C. Netzer, Linda Rausch, Arn H. Eliasson, Hannes Gatterer, Matthias Friess, Martin Burtscher, Stephan Pramsohler

**Affiliations:** ^1^Department of Sport Science, University InnsbruckInnsbruck, Austria; ^2^Department for Hypoxia Research, Hermann Buhl Institute for Hypoxia and Sleep Medicine ResearchBad Aibling, Germany; ^3^Division Sports Medicine and Rehabilitation, Department of Medicine, University UlmUlm, Germany; ^4^Department of Medicine, Uniformed Services University of the Health SciencesBethesda, MD, USA

**Keywords:** normobaric hypoxia, hypobaric hypoxia, altitude, heart rate, oxygen saturation

## Abstract

**Rationale:** Exposures to simulated altitude (normobaric hypoxia, NH) are frequently used in preparation for mountaineering activities at real altitude (hypobaric hypoxia, HH). However, physiological responses to exercise in NH and HH may differ. Unfortunately clinically useful information on such differences is largely lacking. This study therefore compared exercise responses between a simulated hike on a treadmill in NH and a similar field hike in HH.

**Methods:** Six subjects (four men) participated in two trials, one in a NH chamber and a second in HH at an altitude of 4,205 m on the mountain Mauna Kea. Subjects hiked in each setting for 7 h including breaks. In NH, hiking was simulated by walking on a treadmill. To achieve maximal similarity between hikes, subjects used the same nutrition, clothes, and gear weight. Measurements of peripheral oxygen saturation (SpO_2_), heart rate (HR) and barometrical pressure (P_B_)/inspired oxygen fraction (F_i_O_2_) were taken every 15 min. Acute mountain sickness (AMS) symptoms were assessed using the Lake-Louise-Score at altitudes of 2,800, 3,500, and 4,200 m.

**Results:** Mean SpO_2_ values of 85.8% in NH were significantly higher compared to those of 80.2% in HH (*p* = 0.027). Mean HR values of 103 bpm in NH were significantly lower than those of 121 bpm in HH (*p* = 0.029). AMS scores did not differ significantly between the two conditions.

**Conclusion:** Physiological responses to exercise recorded in NH are different from those provoked by HH. These findings are of clinical importance for subjects using simulated altitude to prepare for activity at real altitude.

**Trial registration:** Registration at DRKS. (Approval No. 359/12, Trial No. DRKS00005241).

## Introduction

The popularity of using normobaric hypoxia (NH) to prepare for hypobaric hypoxia (HH) experienced during mountaineering activities at real altitude raises the question about the comparability between the effects of NH and HH. The primary condition that differs between NH and HH is the mechanism by which partial pressure of inspired oxygen (P_i_O_2_) is reduced, with P_i_O_2_ defined as inspired oxygen fraction (FiO_2_) × (P_B_-47 mmHg). Reductions in P_i_O_2_ can be achieved by altering the barometric pressure (P_B_) in HH or the inspired fraction of oxygen (F_i_O_2_) in NH (Conkin and Wessel, 2008). Since seminal work by Paul Bert in 1878, lower P_i_O_2_ has been assumed to be the primary stimulus for adapting to any hypoxic state (Bert, 1878; Conkin and Wessel, 2008). More recently, a direct relationship between lower arterial partial pressure of carbon dioxide (PaCO_2_) and the reduction of P_B_ was found, proposing a synergistic effect of environmental pressure and F_i_O_2_ (Saltzman et al., 1971; Savourey et al., 2003). This effect has been used to explain differences regarding the physiological responses in HH compared to NH environments (Tucker et al., 1983; Roach et al., 1996; Loeppky et al., 2005).

Peripheral oxygen saturation (SpO_2_) and arterial blood oxygen saturation (SaO_2_) have been found to be significantly lower in HH compared to NH. This has been speculated to be due to increased dead space ventilation, provoked by a lower tidal volume (V_t_) and a higher breathing frequency (*f*) (Savourey et al., 2003, 2007). Accordingly, PaCO_2_ decreases with alkalemia as a consequence, shifting the oxyhemoglobin dissociation curve to the left (Savourey et al., 2003). A recent review describes the variations in ventilation in NH and HH (Coppel et al., 2015). The heart rate (HR) is increased in HH (Savourey et al., 2007; Self et al., 2011; Faiss et al., 2013). The severity of acute mountain sickness (AMS) as a result of physiological differences in HH appears to be greater as well (Tucker et al., 1983; Roach et al., 1996; Loeppky et al., 2005).

After years of altitude research most differences between NH and HH appear to be sufficiently explored.

However, one aspect that is still debated is the interchangeability of the HH and NH environments regarding their effects on physiological responses. Previous studies often lack standardization or detailed documentation and do not provide comparability of study designs (Millet et al., 2012a,b; Mounier and Brugniaux, 2012; Saugy et al., 2016). Most of the studies conducted used laboratory settings for both NH and HH (Millet et al., 2012a) Since the advent of chamber research on hypoxia, questions have remained regarding the comparability between conditions of the simulated chamber environment and the real-life mountain environment.

According to a recent review, several studies have compared NH and HH but the comparison of laboratory (NH) and field (HH) environments in a single standardized study has not yet been performed (Coppel et al., 2015). To show differences in SpO_2_, HR, and AMS between laboratory NH and field HH conditions we conducted measurements of these parameters during a hike in simulated altitude and again in real altitude, standardizing variables as much as possible to allow for maximal comparability over the course of a 7-h exposure time. We hypothesized that we could verify the significant differences in SpO_2_, HR, and AMS previously reported by other researchers from laboratory research and research at fixed altitudes by performing a study protocol with continuously increasing altitude in the field.

## Methods

### Subjects

Six healthy participants who usually resided at an altitude between 500 and 650 m (4 men and 2 women, ages 24–45 years) were recruited for the study. They were students of the Department of Sports Science of the University of Innsbruck and reported a moderate fitness routine of 1–4 h per week of training in various disciplines. Descriptive characteristics of participants are shown in Table 1. All subjects gave their written informed consent prior to the participation in the study. None of the participants had been at an altitude above 2,000 m for 6 months prior to the study.

**Table 1:** **Subjects' Characteristics**.

**Subject**	**Sex (m/f)**	**Age (yr)**	**Height (cm)**	**Weight (kg)**	**Weight + backpack (kg)**	**BMI (kg/m^2^)**
1	f	24	169	66	73	23.1
2	m	25	180	62	76	19.1
3	f	26	174	68	71	22.4
4	m	25	174	72	84	23.8
5	m	25	189	82	91	22.9
6	m	45	175	75	81.4	24.5
Mean Values ± *SD*		28.3 ± 8.2	176.8 ± 6.9	70.8 ± 7.1	79.2 ± 7.5	22.6 ± 1.9

### Protocol

Participants completed two trials (T_1_ and T_2_). T_1_ took place in a normobaric hypoxia chamber. Normobaric hypoxia was provided by an oxygen expulsion system (Low Oxygen Systems; Berlin-Buch, Germany). The expulsion system (oxygen exchange through nitrogen) allows a mixture of fresh air to keep controlled CO_2_ levels comparable to the levels measured in HH. Four weeks after the first trial, participants went through an equivalent testing protocol (T_2_) under hypobaric hypoxia conditions on the mountain Mauna Kea, Hawaii, USA. Mauna Kea was chosen for the experiment for a number of important reasons. The mountain's altitude with a moderate continuous inclination allowed the simulated treadmill hike to be programmed to a high degree of comparability. Constant climatic conditions on the mountain with temperatures around 20°C paralleled the temperatures in the normobaric hypoxia room. Road access to the summit facilitated transport of gear and descent of the participants. Participants were asked to wear the same clothes and use the same equipment during each trial. The amount of food and beverage intake was weighed and recorded for each person at breakfast and dinner before T_1._ The subjects were instructed to follow the same food and beverage intake in T_2_. Participants carried the same weight consisting of snacks and beverage in a backpack during both trials. Energy and fluid intake were measured individually during T_1_ and kept the same for T_2_. Temperature and humidity were continually measured in both settings.

### T_1_ protocol

The first trial in NH conditions (T_1_) involved a 7-h stay in the chamber with 6 h walking on a treadmill (h/p Cosmos Mercury and Quasar). The built-in calibrating sensor of the treadmills were checked for accuracy by a mechanic prior to testing (error < 0.2 m per 100-m interval). The treadmill hike was set at an average incline of 14.2% and a constant speed of 1.6 km/h. These parameters were calculated to simulate the hike on top of the Mauna Kea Mountain. The average incline was determined as the ratio between the difference in altitude (1,405 m) and the walking distance (9,894 m). The treadmill speed was calculated as a function of the duration of walking (6 h) and the walking distance. Participants started walking at a simulated altitude of 2,800 m. The F_i_O_2_ in the chamber was modified automatically, which led to the completion of the treadmill task at a simulated altitude of 4,200 m. During the testing, participants had 3 breaks; a 15-min break after 75 min of walking, a 30-min break after 195 min of walking and another 15-min break after 315 min of walking. During the breaks, participants stayed in the chamber, resting, drinking, and eating snacks. An investigator was present throughout all testing in NH, controlling the simulated altitude, time, walking distance, and F_i_O_2_ (measured by O_2_ and CO_2_ probes in the room, Dräger Germany).

### T_2_ protocol

In the second trial, participants arrived on the Big Island Hawaii and rested for 4 days after arrival with at least 8 h of sleep per night. Participants then hiked the Humu'ula trail on top of the Mauna Kea Mountain. The subjects were brought to the starting point of the hike via a 30-min car drive. The experiment started at 09:00 AM local lime. The trail starts at an altitude of 2,800 m and ends at 4,200 m. Participants used a GPS receiver (Forerunner® 305, Garmin, Garching, Germany) to measure time and walking distance. The PM 90 monitor (Beurer GmbH, Ulm, Germany) was used to evaluate barometric pressure and altitude as well as to keep a constant walking speed. We report each 15-min measurement interval as 50 m in altitude gain. Two 15-min breaks and one 30-min break were made after the same time of walking as in T_1_. Participants rested, ate snacks and drank fluids during the breaks just as in T_1_.

### Measurements

Anthropometric measurements were made and participants were weighed with and without their backpacks before T_1_. For at least 1 min every 15 min during the hike, measurements of HR and SpO_2_ were conducted by the participants themselves. The measurements were done using fingertip pulse oximeters (PULOX® PO-100, Novidion GmbH, Cologne, Germany, accuracy for SpO_2_ = 70–100 ± 2% and for HR = 30–250 ± 2 beats per min). Participants avoided solar radiation on the devices and noted the stable SpO_2_ at the end of the 1 min measurement (Luks and Swenson, 2011). Self-reported recordings were visually checked by one investigator and one other participant independently for possible artifacts. Only one value, a SpO2 in subject 5, had to be corrected. The rate of perceived exertion was assessed via Borg scale directly after the hike.

Subjective symptoms of AMS were assessed using the Lake-Louise-Score (LLS) Questionnaire at altitudes if 2,800, 3,500, and 4,200 m (Roach et al., 1993). The following AMS categories were assessed: (1) headache, (2) gastrointestinal symptoms (e.g., nausea), (3) fatigue and/or weakness, (4) dizziness/lightheadedness. According to the literature, the 5th symptom complex of the LLS, i.e., the quality of sleep, was excluded from the self-report section (Macinnis et al., 2013). Participants rated the severity of AMS symptoms from 0 (no discomfort) to 3 (severe discomfort). AMS was diagnosed when headache occurred together with one other symptom from the categories mentioned above, and a total LLS of >3 (Hackett and Roach, 2001). Walking time, distance, speed, and altitude were assessed the same way in T_1_ and T_2_.

### Statistics

Although sample size was primarily based on logistical considerations, a power analysis was performed with G-Power Version 3.1.9.2 (University of Kiel) for dependent samples (correlation coefficient set at 0.5) with expected values of mean SpO_2_ 90 ± 3% for NH and 85 ± 3% for HH based on previous study results (Savourey et al., 1997, 2003, 2007; Boos et al., 2016). The power analysis yielded a sample size of six subjects. Statistical analysis was performed using IBM SPSS Statistics 23 (PASW Statistics for Windows version 21.0, SPSS Inc., Chicago, IL, USA). Data were expressed as means and standard deviations or as medians with 95% confidence intervals as appropriate. Nonparametric tests (Wilcoxon) were used for HR, SpO_2_, and AMS scores at each time point comparing NH and HH. The significance threshold was set at 0.05.

### Study approval

The Ethics Committee at the Leopold-Franzens-University of Innsbruck, Austria approved the study protocol on the October 1, 2014. Participants gave their written informed consent prior to inclusion in the study.

## Results

**Figure 1:**
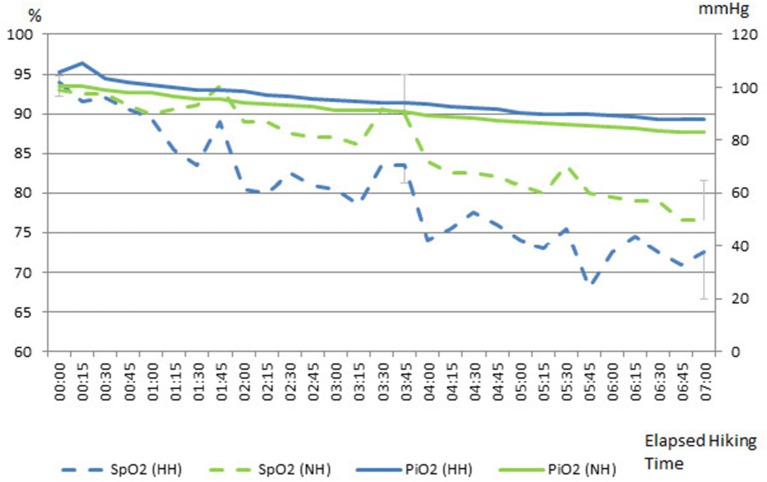
**Comparison of inspired oxygen (P_**i**_O_**2**_) and peripheral oxygen saturation (SpO_**2**_) during the hikes in normobaric hypoxia (NH) and hypobaric hypoxia (HH)**. Legend: Break 1 at 3,100 m altitude, Break 2 at 3,500 m altitude, Break 3 at 3,900 m altitude; % = SpO_2_%; mmHg = P_i_O_2_ mmHg.

All participants completed the two exposure trials successfully. During ascent in NH, F_i_O_2_ was lowered continuously from 14.6% at 2,800 m, 13.2% at 3,500 m to 12.1% at 4,205 m (P_B_ = 767.3 mmHg). The room air CO_2_ concentration was kept relatively constant during the experiment at 884 ppm ± 325 within limits that did not influence ventilation. On the mountain, P_B_ was measured with a value of 550 mmHg at 2,800 m, 496 mmHg at 3,500 m and 465 mmHg at 4,205 m (Figure 1). Temperature ranged in both conditions (at Mauna Kea between sunrise and sunset) at all altitude levels around 20°C (19–24°C) and humidity was between 50 and 60% (average on Mauna Kea 58%; in the chamber 55%). Wind speed on Mauna Kea was below 5 mph.

An overall statistical difference between NH and HH regarding HR and SpO_2_ was detected (*p* < 0.05; Tables 2, 3). However, AMS scores did not differ significantly between the chamber and the mountain. Self-reported Borg scale values showed no difference (*p* = 1.00) for rate of perceived exertion in both NH (12.33 ± 2.07) and HH (12.33 ± 1.75).

**Table 2:** **Actual fraction of inspired oxygen (F_**i**_O_**2**_) in normobaric hypoxia (NH) and barometric pressure (P_**B**_) in hypobaric hypoxia (HH). Medians (confidence intervals) of heart rate (HR) and ***p***-values between NH and HH**.

**F_i_O_2_ %**	**P_B_ mmHg**	**Altitude (m)**	**HR median (bpm) and 95% CI**		**NH vs. HH**
			**NH**	**HH**	***p*****-value**
14.57	550	2,800	93 (77–109)	87 (72–102)	0.225
14.57	566	2,850	108 (100–115)	123 (109–137)	0.028
14.33	539	2,900	104 (93–114)	110 (93–126)	0.249
14.25	533	2,950	101 (91–111)	122 (101–143)	0.046
14.21	527	3,000	102 (89–114)	120 (104–136)	0.027
14.02	523	3,050	103 (91–114)	125 (109–140)	0.027
13.89	518	3,100	103 (88–118)	122 (112–131)	0.027
13.85	518	3,150	84 (69–99)	94 (76–111)	0.080
13.66	516	3,200	103 (92–114)	131 (114–148)	0.028
13.56	509	3,250	104 (95–113)	122 (105–139)	0.028
13.50	506	3,300	105 (94–116)	122 (96–148)	0.028
13.46	503	3,350	104 (94–114)	125 (107–142)	0.027
13.24	500	3,400	103 (90–115)	129 (108–149)	0.028
13.24	497	3,450	105 (93–116)	133 (112–153)	0.028
13.21	496	3,500	80 (62–98)	87 (68–105)	0.115
13.18	496	3,550	75 (59–90)	87 (69–105)	0.046
12.97	493	3,600	107 (95–119)	130 (96–164)	0.173
12.91	489	3,650	111 (97–124)	131 (114–148)	0.027
12.80	486	3,700	109 (91–126)	135 (105–165)	0.046
12.69	483	3,750	109 (92–126)	128 (115–141)	0.028
12.63	478	3,800	111 (93–128)	136 (120–152)	0.027
12.52	476	3,850	110 (93–127)	138 (118–158)	0.028
12.49	476	3,900	87 (67–107)	94 (77–111)	0.058
12.37	476	3,950	112 (94–129)	126 (91–161)	0.173
12.33	473	4,000	113 (95–131)	122 (108–136)	0.115
12.26	470	4,050	115 (98–132)	115 (96–133)	0.345
12.10	467	4,100	121 (106–135)	125 (106–144)	0.173
12.08	465	4,150	114 (93–134)	132 (107–157)	0.046
12.03	465	4,200	114 (92–135)	130 (105–155)	0.046
		Mean	103 (93–114)	121 (106–135)	

**Table 3:** **Fractions of inspired oxygen (F_**i**_O_**2**_) in normobaric hypoxia (NH), barometric pressure (P_**B**_) in hypobaric hypoxia (HH), medians (confidence intervals) of peripheral oxygen saturation (SpO_**2**_) and ***p***-values between NH and HH**.

**F_i_O_2_ %**	**P_B_ mmHg**	**Altitude (m)**	**S_p_O_2_ median (%) and 95% CI**		**NH vs. HH**
			**NH**	**HH**	***p*****-value**
14.57	550	2,800	93 (91–95)	94 (92–96)	0.336
14.57	566	2,850	93 (91–94)	92 (88–95)	0.399
14.33	539	2,900	93 (90–95)	92 (89–95)	0.248
14.25	533	2,950	91 (89–93)	91 (85–96)	0.786
14.21	527	3,000	90 (88–92)	90 (85–94)	0.854
14.02	523	3,050	91 (86–95)	86 (83–88)	0.078
13.89	518	3,100	91 (85–97)	84 (79–88)	0.027
13.85	518	3,150	94 (90–97)	89 (86–92)	0.027
13.66	516	3,200	89 (84–94)	81 (75–86)	0.028
13.56	509	3,250	89 (85–93)	80 (78–82)	0.026
13.50	506	3,300	88 (83–93)	83 (78–87)	0.026
13.46	503	3,350	87 (82–92)	81 (75–87)	0.058
13.24	500	3,400	87 (82–92)	81 (75–86)	0.172
13.24	497	3,450	86 (81–91)	79 (72–85)	0.046
13.21	496	3,500	91 (88–93)	84 (79–89)	0.028
13.18	496	3,550	90 (86–94)	84 (75–92)	0.075
12.97	493	3,600	84 (79–89)	74 (68–80)	0.027
12.91	489	3,650	83 (77–88)	76 (71–81)	0.027
12.80	486	3,700	83 (78–88)	78 (72–83)	0.112
12.69	483	3,750	82 (77–87)	76 (70–82)	0.027
12.63	478	3,800	81 (75–87)	74 (70–78)	0.027
12.52	476	3,850	80 (75–85)	73 (64–82)	0.046
12.49	476	3,900	84 (79–88)	76 (64–87)	0.028
12.37	476	3,950	80 (74–86)	68 (61–75)	0.027
12.33	473	4,000	80 (73–86)	73 (71–74)	0.046
12.26	470	4,050	79 (74–84)	75 (70–80)	0.058
12.10	467	4,100	79 (74–84)	73 (67–78)	0.027
12.08	465	4,150	77 (71–82)	71 (64–78)	0.027
12.03	465	4,200	77 (71–82)	73 (68–78)	0.027
		Mean	86 (82–90)	80.2 (77–83)	

### Heart rate

**Figure 2:**
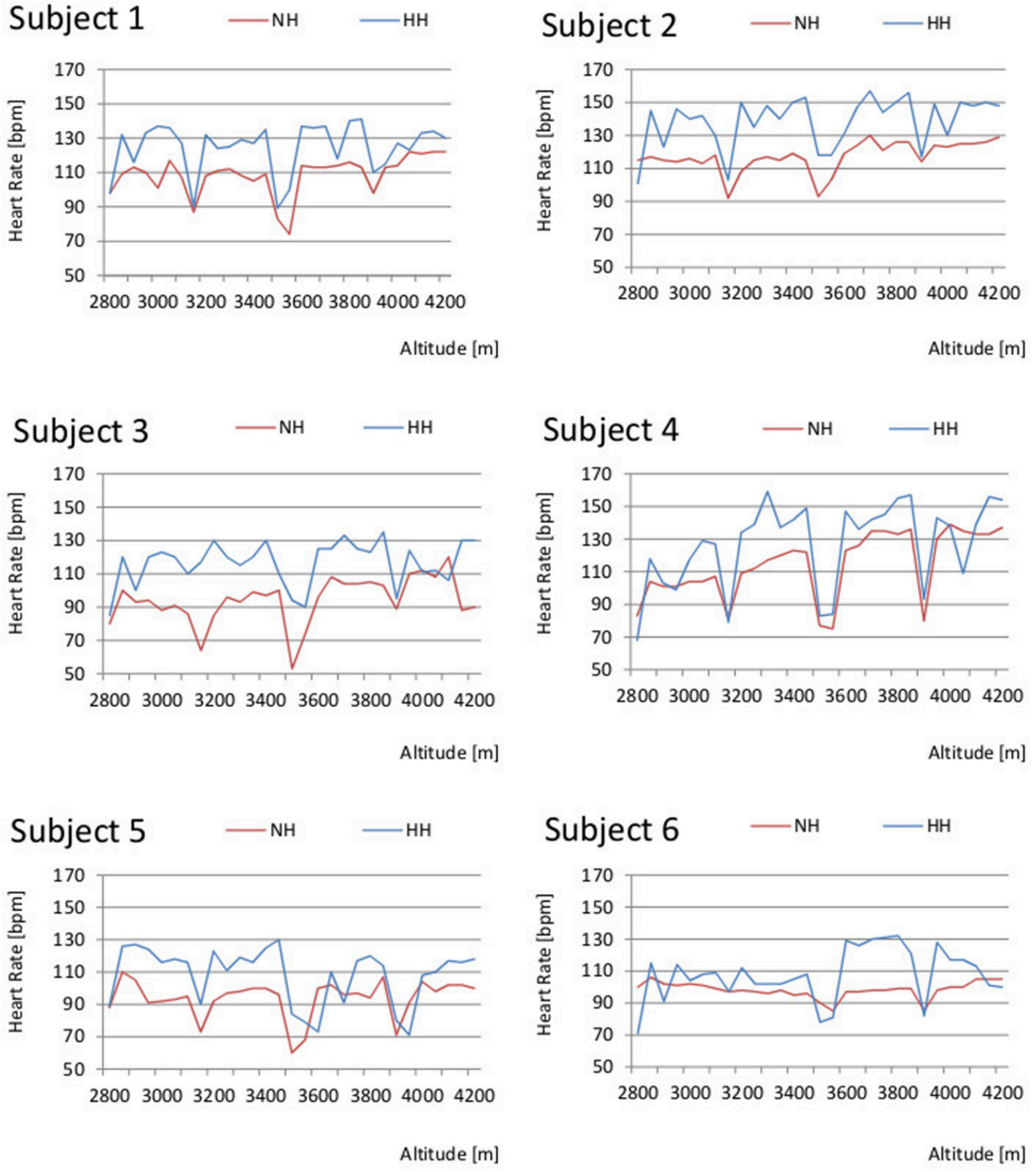
**Comparison of individuals' heart rate (HR) in normobaric hypoxia (NH) and hypobaric hypoxia (HH)**.

The HR showed significant differences in 66% of the measured time points between NH and HH (*p*-values varied from *p* = 0.027–0.046, Table 2). In both NH and HH, the lowest values of individual HR were observed during or after the three walking breaks around 3,100, 3,500, and 3,900 m (Figure 2). Minimum HR values ranged from 53 to 93 beats per min (bpm) in NH and from 68 to 103 bpm in HH. Peak values extended from 106 to 137 bpm in NH and from 130 bpm to 159 in HH.

### Peripheral oxygen saturation

**Figure 3:**
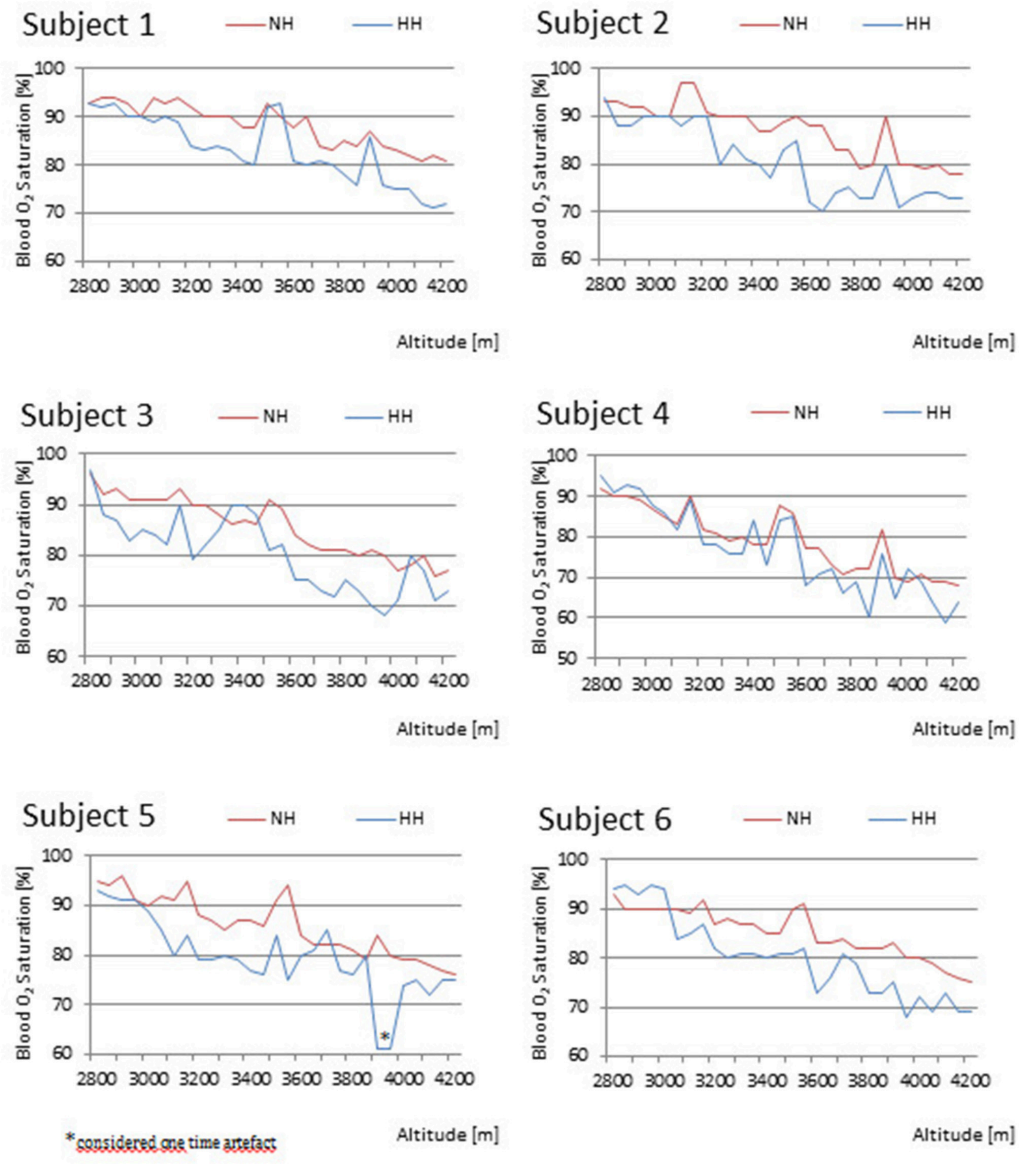
**Comparison of individual's peripheral oxygen saturation (SpO_**2**_) between normobaric hypoxia (NH) and hypobaric hypoxia (HH)**.

Significant differences in SpO_2_ were seen between NH and HH in 62% of the measured time points (Table 3). Most significant *p*-values for SpO_2_ were detected (*p*-values ranged from *p* = 0.027–0.046) from 3,100 to 3,300 m and from 3,750 to 4,000 m. The SpO_2_ values for each participant decreased with increasing altitude (Figure 3). Highest values of SpO_2_ were reached during or after the 3 breaks around 3,100, 3,500, and 3,900 m. Peak values ranged from 95 to 97% in NH and from 93 to 95% in HH. Lowest SpO_2_ values ranged from 68 to 82% in NH and from 61 to 75% in HH.

In the two women (subjects 1 and 3), SpO_2_ in HH was higher compared to NH around the break at 3,500 m with values ranging from 90 to 93% in HH compared to 88 and 91% in NH. Subjects 2 and 6 were the only participants in whom the SpO_2_ values in HH were lower throughout the whole protocol except for the beginning at 2,800 m. In subject 6, the SpO_2_ values stayed higher in HH up to 3,050 m. Subject 5 randomly showed one higher SpO_2_ value of 85% in HH compared to 82% in NH at 3,700 m. Therefore, we consider the one time measurement of 61% at 3,950 m an artifact. The largest difference in SpO_2_ values was found in subject 4 varying from 95% at 2,800 m to 60% at 3,850 and 4,150 m in HH.

### Acute mountain sickness

There was no significant difference in AMS scores examined (*p* > 0.05) between NH and HH. The severity of AMS was mild (LLS 3 to 4) in almost all subjects in both NH and HH. On average, median AMS scores were lower than 3, except for the increased median value of 3.5 at an altitude of 4,200 m in HH which is not statistically significant. Half of the subjects reported an AMS severity of 4 at 4,200 m in HH, whereas one participant suffered from mild AMS (LLS = 4) at a lower altitude of 3,500 m on the mountain. Two subjects experienced a LLS ≥ 3 in NH. None of the participants noted severe nausea and vomiting or severe fatigue or dizziness.

## Discussion

This is the first study to compare a 7-h simulated high altitude hike in a normobaric hypoxia chamber and a mountain hike at real altitude. This study provides a descriptive comparison of physiological parameters (HR and SpO_2_) and AMS scores measured in a chamber (NH) and on the mountain (HH). Our findings do verify previously reported differences for SpO_2_ and HR, with SpO_2_ values significantly lower and HR values significantly higher in a HH environment compared to NH. However, in contrast to prior reports, AMS severity did not differ significantly between the two settings.

Using a study design similar to our protocol, Savourey et al. also found SpO_2_ to be significantly lower in a hypobaric chamber (HH) compared to NH with a simulated altitude exposure to 4,500 m (Savourey et al., 2003). The Savourey study was performed at a fixed altitude for a limited time only. However, ventilation was measured and revealed increased dead space ventilation in HH due to a lower tidal volume (V_t_) and a higher frequency of breathing (*f*). This breathing pattern caused a lower PaCO_2_, alkalemia, and a lower SpO_2_ than in NH (Savourey et al., 2003). Other studies reported a direct relationship between a lower PaCO_2_ and the reduction of P_B_ (Saltzman et al., 1971). To explain the findings of significantly lower SaO_2_ values in HH compared to NH at a simulated altitude of 7,620 m a synergistic effect of hypoxia and hypobaria was proposed (Levine et al., 1988; Self et al., 2011).

The studies cited above were characterized by a short exposure time to hypoxia (<1 h) at fixed altitudes. The importance of exposure time was emphasized in a review of five long-term hypoxia exposure studies (>1 h) which did not show any differences in SpO_2_ between NH and HH,(Tucker et al., 1983; Roach et al., 1996; Loeppky et al., 1997, 2005; Faiss et al., 2013) possibly due to acclimatization to hypoxia (Coppel et al., 2015). What sets our study apart from prior research is that the study design incorporated a long exposure time at increasing altitudes with movement and exercise performance utilizing actual mountaineering conditions.

Subjects 1 and 3 reacted with values ranging from 90 to 93% in HH compared to 88 and 91% in NH around the 3,500 m break. Because both participants were women, a likely explanation of this finding may be increased respiratory drive due to higher progesterone levels in these participants.

In previous short-term as well as in long-term exposure studies, heart rate values tended to be higher in HH, but showed inconsistent results compared to NH (Savourey et al., 1997). In their first study, Savourey et al. recorded HR values that were significantly higher in HH, whereas in their second study no significant differences in HR were found, despite using a very similar protocol (Savourey et al., 2003, 2007). While the subjects of Savourey et al. were exposed to altitude for only 40 min, Faiss et al. conducted a 24-h exposure study at 3,000 m and did not find any significant differences in HR at rest or during moderate physical activity between conditions of NH and HH (Faiss et al., 2013). In our study HR was significantly higher in HH for the duration of 7 h. The increased HR values may be explained by a need for higher cardiac output due to a lower minute ventilation (V_E_) from faster and shallower breathing in HH as has often reported in studies (Savourey et al., 2007; Self et al., 2011; Faiss et al., 2013).

There are other possible explanations for the increased HR we demonstrated during the mountain trail hike: (a) the ground surface on the Humu'ula trail is uneven compared to a treadmill and could have elicited higher exercise performance, (b) ultraviolet light radiation of the sun could have led increased fluid loss on the mountain trail compared to the chamber conditions. With restrictions on fluid intake to maintain comparability of intake between study settings, volume depletion with consequent tachycardia may have occurred, and (c) participants reported marked differences in group dynamics between the settings with more group pressure on Mauna Kea.

Our finding regarding the lack of difference in severity of AMS symptoms between NH and HH agrees with previous research (Richard et al., 2014). There are two factors that affect the generalizability of this finding. First, there was high inter-subject variability with the LLS probably due to the subjective nature of this questionnaire. Second, AMS becomes fully manifest after 6–96 h of hypoxic exposure at moderate to high altitudes (Debevec and Millet, 2014) Therefore, the 7-h exposure duration used in our study design may have had an impact on the participants' experiences of AMS during NH and HH. Limited exposure duration may explain the discrepancies between our observations and those studies reporting the opposite finding with exposure durations longer than 8 h (Debevec and Millet, 2014).

As is true of all studies with high altitude field observations, our study has limitations. Due to logistical problems, it was not possible to randomize assignments to the different study stages. It was also not possible to blind subjects to the experimental conditions in this crossover trial comparing NH in a chamber with natural HH on a mountain. However, due to the fact that the study results are based predominantly on objective parameters as SpO_2_ and heart rate the likelihood of influencing results with personal bias is low. A minor limitation is the potential confounding effect of hypobaric acclimatization during the flights to Hawaii (10 and 5 h with 14 h lay-over between flights) as well as the jetlag effect on circadian rhythms. However, 4 days of rest at sea level on Hawaii with extended sleep times and time for sun exposure on the beach diminished these potential cofounding effects (Reilly and Edwards, 2007). Another minor limitation is that we did not measure urine concentrations after the hikes such that we cannot determine the possible contribution of a greater degree of volume depletion during the mountain hike.

In conclusion, our study describes physiological differences in HR and S_p_O_2_ during a mountain hike at altitudes from 2,800 to 4,200 m compared to a simulated hike in a normobaric hypoxia chamber. These differences must receive consideration when high altitude studies are performed or when mountaineers train for hikes at high altitude using normobaric chambers. The results of our trial also provide important data for aviation medicine and intensive care medicine, environments that confront the critical issue of hypoxia.

## Author contributions

NN: designing research study, conducting experiment, providing material, writing manuscript LR: conducting experiment, writing manuscript, acquiring data, analyzing data Manuscript AE, Writing final version of manuscript; HG, analyzing data, writing manuscript; MF, conducting experiment, acquiring data; MB, writing manuscript, analyzing data; SP, designing research study, conducting experiment, analyzing data, acquiring data, writing.

### Conflict of interest statement

The authors declare that the research was conducted in the absence of any commercial or financial relationships that could be construed as a potential conflict of interest.
